# Enhancing Hybrid Prediction in Pearl Millet Using Genomic and/or Multi-Environment Phenotypic Information of Inbreds

**DOI:** 10.3389/fgene.2019.01294

**Published:** 2020-01-24

**Authors:** Diego Jarquin, Reka Howard, Zhikai Liang, Shashi K. Gupta, James C. Schnable, Jose Crossa

**Affiliations:** ^1^ Department of Agronomy and Horticulture, University of Nebraska-Lincoln, Lincoln, NE, United States; ^2^ Department of Statistics, University of Nebraska-Lincoln, Lincoln, NE, United States; ^3^ International Crops Research Institute for the Semi-Arid Tropics (ICRISAT), Hyderabad, India; ^4^ International Maize and Wheat Improvement Center (CIMMYT), Ciudad de Mexico, Mexico

**Keywords:** genomic selection, hybrid prediction, genotype-by-environment interaction G×E, general combining ability, specific combining ability, conventional and tunable GBS

## Abstract

Genomic selection (GS) is an emerging methodology that helps select superior lines among experimental cultivars in plant breeding programs. It offers the opportunity to increase the productivity of cultivars by delivering increased genetic gains and reducing the breeding cycles. This methodology requires inexpensive and sufficiently dense marker information to be successful, and with whole genome sequencing, it has become an important tool in many crops. The recent assembly of the pearl millet genome has made it possible to employ GS models to improve the selection procedure in pearl millet breeding programs. Here, three GS models were implemented and compared using grain yield and dense molecular marker information of pearl millet obtained from two different genotyping platforms (C [conventional GBS RAD-seq] and T [tunable GBS tGBS]). The models were evaluated using three different cross-validation (CV) schemes mimicking real situations that breeders face in breeding programs: CV2 resembles an incomplete field trial, CV1 predicts the performance of untested hybrids, and CV0 predicts the performance of hybrids in unobserved environments. We found that (*i*) adding phenotypic information of parental inbreds to the calibration sets improved predictive ability, (*ii*) accounting for genotype-by-environment interaction also increased the performance of the models, and (*iii*) superior strategies should consider the use of the molecular markers derived from the T platform (tGBS).

## Introduction

Pearl millet [*Cenchrus americanus* (L.) Morrone; Syn. *Pennisetum glaucum* (L.) R. Br.] is a heat and drought tolerant grain and forage crop widely cultivated in South Asia and sub-Saharan Africa. Production in West Africa relies primarily on open-pollinated varieties (OPV), while in South Asia hybrid production based on cytoplasmic male sterility has been widely adopted. Crop improvement efforts in pearl millet face three primary challenges: (*i*) efficiently breeding new elite parental lines when inbred phenotypes are unreliable proxies for hybrid performance; (*ii*) balancing trade-offs between selecting for traits associated with optimal forage yield and quantity and those associated with optimal grain yield and grain quality; and (*iii*) significant genotype-by-environment (G×E) interactions driving inconsistent response patterns. In many parts of the world, pearl millet is planted during or immediately after a rainy season, and the maximum growing season is closed by terminal drought. Hence, matching flowering time and drought avoidance strategies between pearl millet OPVs or hybrids and locations is critical to maximizing yield potential.

The genome of pearl millet was recently assembled and released (Varshney et al., 2017). The availability of a reference genome has made it practical to begin employing genomic selection to predict both the general combining ability (GCA) of new inbred parents, and the specific performance of particular pearl millet hybrids (Liang et al., 2018). It is shown that incorporating parental information into genomic prediction can be beneficial (Massman et al., 2013). Sequencing-based genotyping enables to generate thousands of SNPs for separating breeding lines in a given population. Two methods of genotyping, RAD-seq (Miller et al., 2007; Baird et al., 2008) and tGBS (Ott et al., 2017), were widely used in breeding programs (Kim et al., 2016; Zheng et al., 2018; Li et al., 2019). RAD-seq can generate more SNP sites compared with tGBS, while SNP sites called by tGBS have higher read depths than RAD-seq.

The incorporation of high-throughput genotyping and estimated breeding values into genomic selection assisted breeding programs have the potential to address all three major bottlenecks of pearl millet breeding efforts outlined above (*i–iii*). Given the diverse climates in which pearl millet hybrids are cultivated, effective and practical genomic prediction models require to explicitly incorporate and model G×E interactions in order to provide breeders with relevant predictions and estimated breeding values.

Here we assess the performance of genomic prediction models for yield pearl millet under several factors: (1) two genotyping platforms (C [conventional GBS] and T [tunable GBS or tGBS]) for sequencing parental inbreds; (2) composition of calibration sets by augmenting these with phenotypic information of inbreds; and (3) effects of accounting for the G×E component.

Three different cross-validation (CV) schemes were simulated for mimicking realistic scenarios that breeders might face in different stages of the breeding pipeline (CV2: predicting tested genotypes in observed locations; CV1: predicting untested genotypes in observed locations; and CV0: predicting tested genotypes in unobserved locations). In all cases, the goal was to predict the performance of hybrids at the trial level (i.e., correlations between predicted and observed values were compute for genotypes within the same environment).

The genetic relationships among hybrids were modeled using inbred marker information of the inbred parents *via* the GCA and the specific combining ability (SCA) components. For the GCA component, the genomic relationship matrices (GRMs) for parent 1 and parent 2 were built using the corresponding marker profiles (Bernardo, 1994; Technow et al., 2014; Kadam et al., 2016). For the SCA approach, the Hadamard product of the inbreds’ GRM was used to model the interaction effect between the parental inbreds. Acosta-Pech et al. (2017) showed how to implements this model. In addition to these models, we also incorporated the interaction of the GCA and SCA components with environments, as shown by Acosta-Pech et al. (2017) and Basnet et al. (2018). In total, three models were implemented for analysis, all of them based exclusively on molecular marker data of the inbred parents (GCA and SCA terms).

In general, the results show the advantages of including the parental inbreds’ phenotypic information in the calibration sets. In most cases, accounting for G×E interaction improved the predictive ability the most. Finally, the optimal prediction strategy should consider the molecular marker information derived from T platform.

## Materials And Methods

### Phenotypic and Genomic Information

The phenotypic (grain yield) and genotypic (C and T platforms) information used in this study were taken from Liang et al. (2018). Grain yield measurements were collected from 320 pearl millet hybrids and 37 inbred parents collected in replicated yield trials across four environments in India in 2015 (Environment 1: Dhule, Environment 2: Hisar, Environment 3: Patancheru, Environment 4: Jamnagar). The inbred parents of each hybrid were genotyped using two platforms: conventional GBS [C platform] (Miller et al., 2007; Baird et al., 2008) and tGBS [T platform] (Ott et al., 2017; Varshney et al., 2017). Both platforms produced SNP sets that had missing data which were imputed using Beagle (Browning et al., 2018) (Version 16-06-2016). Initially, 649,067 polymorphic SNPs were identified using the C platform while 73,291 were identified for the T platform. After the SNPs with minor allele frequency (MAF) < 0.05 were excluded the platform C scored 28,495 SNPs while the platform T scored 30,222 SNPs. While absolute costs per sample can vary significantly based on labor and reagent costs, platform C required an average of 12.2M reads per sample and platform T required an average of 1.8M reads per sample suggesting that costs is either comparable (when the cost of labor predominates) or lower for platform T (when the cost of sequencing predominates).

After the aligning the phenotypic and genomic data (for both platforms) considering only common genotypes observed in all environments, the number of unique hybrids and inbreds remaining in the analysis were 276 and 33, respectively. The 33 inbreds correspond to two non-overlapped sets of parents: 20 acting exclusively as parent 1 (P1, B-lines) and the remaining 13 acting as parent 2 (P2, R-lines). Detailed methodologies for trait collection and genotype calling are described in Liang et al. (2018).

### Genomic Prediction Models

#### M1. General Combining Ability (GCA) Model (E + G_P1_ + G_P2_)

For charactering the *i* ^th^ hybrid, this model uses the genomic information from the inbreds *via* the GCA of the parents; thus, the male and female effects can be modeled. This model is composed by two genetic scores, which are derived from the main effects of the markers of those inbreds acting as parent 1 or B-lines (*g*_*P*1*i*_) and parent 2 or R-lines (*g*_*P*2*i*_), respectively. In the model $g_{P1i}=\overset{p}{\underset{m=1}{\sum }}x_{P1im}b_{m}$ and $g_{P2i}=\overset{p}{\underset{m=1}{\sum }}x_{P2im}b_{m}$ are the genetic effects modeled as the linear combination between *p* marker covariates (*x*_*im*_) and their corresponding marker effects (*b*_*m*_) for *m* = 1, 2,…, *p* with $b_{m}\overset{iid}{∼}N\left(0,\sigma_{b}^{2}\right)$ and $\sigma_{b}^{2}$ acting as the common marker effect variance acting as the common marker effect variance; *iid* stands for independent and identically distributed. *X*_*P*1_, *X*_*P*2_ (with dimensions of 20 × 28,495 for platform C and 20 × 30,222 for platform T, and 13 × 28,495 for platform C and 13 × 30,222 for platform T, respectively) are the corresponding marker matrices of the inbreds (acting as parent 1 and parent 2, respectively) involved in the hybrid genotypes (Bernardo, 1994; Technow et al., 2014; Kadam et al., 2016) and these contain the number of copies of the major allele for each inbred at each marker position such that *x*∈{0, 2}. Collecting the aforementioned results and assumptions, the linear predictor for modeling the hybrid performance *via* the GCA of inbreds is obtained as follows

(1) $$y_{ij}=\mu +E_{j}+{\text{g}}_{P1i}+{\text{g}}_{P2i}+e_{ij}$$

where *y*_*ij*_ is the yield performance of the *i* ^th^ (*i* = 1, 2, …, *I*) hybrid in the *j* ^th^ (*j* = 1, 2,…,*J*) environment*, μ* is the common mean, *E*_*j*_ is the main effects of the *j* ^th^ environments such that $E_{j}\overset{iid}{∼}N\left(0,\sigma_{E}^{2}\right)$, $g_{P1}=\left\{{\text{g}}_{P1i}\right\}∼N\left(0,G_{P1}\sigma_{P1\text{g}}^{2}\right)$ and $g_{P2}=\left\{{\text{g}}_{P2i}\right\}∼N\left(0,G_{P2}\sigma_{P2\text{g}}^{2}\right)$ with $G_{P1}=\frac{X_{P1}X_{P1}^{\prime }}{p}$, $G_{P2}=\frac{X_{P2}X_{P2}^{\prime }}{p}$, $\sigma_{P1\text{g}}^{2}=p\times \sigma_{bP1}^{2}$ and $\sigma_{P2\text{g}}^{2}=p\times \sigma_{bP2}^{2}$ as the corresponding variance components of the parental effects, and $e_{ij}\overset{iid}{∼}N\left(0,\sigma_{e}^{2}\right)$ and $\sigma_{E}^{2}$, and $\sigma_{e}^{2}$ represent the associated variance components of environments, and residual terms. One of the disadvantages of this model is that it does not take into consideration the specific effect of crossing parent 1 with parent 2, but rather the average effects between both parents. Moreover, it returns a common genetic effect for the same hybrid in different environments. 

#### M2. General Plus Specific Combining Ability Model (E + G_P1_ + G_P2_ + G_P1 × P2_)

This model is an extension of model M1, and it not only accounts for the main effects of the genetic components of the inbreds, but also includes the specific interaction effect of crossing inbred parent 1 and parent 2 (Acosta-Pech et al., 2017). The main effect is accounted for by the GCA component, and the interaction effect is accounted for by the SCA component. The SCA was modeled using the cell-by-cell product of the entries of the co-variance structures from inbred parent 1 (***G***_*P*1_) and inbred parent 2 (***G***_*P*2_), such that $g_{P1\times P2}=\left\{{\text{g}}_{P1i\times P2i}\right\}∼N\left(0,G_{P1\times P2}\sigma_{P1\text{g}\times P2\text{g}}^{2}\right)$, where $G_{P1\times P2}=\;\left(Z_{gP1}G_{P1}Z_{gP1}^{\prime }\right){}^{\circ}\left(Z_{gP2}G_{P2}Z_{gP2}^{\prime }\right)$, $\sigma_{P1\text{g}\times P2\text{g}}^{2}$ is the variance component associated with this interaction term, and ***Z***_*gP*1_ and ***Z***_*gP*2_ are the corresponding incidence matrices for parent 1 and parent 2 for the hybrids such that these are of order 276 × 20 and 276 × 13 for the case when no phenotypic inbred data was included in calibration sets and of 309 (276 + 33) × 20 and 309 (276 + 33) × 13 when augmenting calibration sets with phenotypic inbred data.

The model in which both the GCA and the SCA components are included can be written as

(2) $$y_{ij}=\mu +E_{j}+{\text{g}}_{P1i}+{\text{g}}_{P2i}+{\text{g}}_{P1i\times P2i}+e_{ij},$$

where all of the terms are defined above. Although this model consider the effects of crossing parent 1 with parent 2 it also return a common genetic effect across environments for same hybrid in different environments similarly to the previous model.

#### M3. General Plus Specific Combining Ability in Interaction With Environments Model (E + G_P1_ + G_P2_ + G_P1 × P2_ + G_P1_ × E + G_P2_ × E + G_P1 × P2_ × E)

This model is an extension of M2, in that it includes both the GCA and SCA components but also accounts for the interaction of the inbred markers with environments by including the interaction between the GCA and SCA components and environments. The model can be written as

(3) $$y_{ij}=\mu +E_{j}+{\text{g}}_{P1i}+{\text{g}}_{P2i}+{\text{g}}_{P1i\times P2i}+{\text{gE}}_{P1ij}+{\text{gE}}_{P2ij}+{\text{gE}}_{P1ij\times P2ij}+e_{ij}$$

where $gE_{P1}=\left\{{\text{gE}}_{P1ij}\right\}∼N\left(0,\left(Z_{gP1}G_{P1}Z_{gP1}^{\prime }\right){}^{\circ}\left(Z_{E}Z_{E}^{\prime }\right)\sigma_{gEP1}^{2}\right)$, $gE_{P2}=\{{\text{gE}}_{P2ij}\}∼N\left(0,\left(Z_{gP2}G_{P2}Z_{gP2}^{\prime }\right){}^{\circ}\left(Z_{E}Z_{E}^{\prime }\right)\sigma_{gEP2}^{2}\right)$ and $gE_{P1xP2}=\left\{{\text{gE}}_{P1ij\times P2ij}\right\}∼N\left(0,\left(I_{4}⊗\left(\left(Z_{gP1}G_{P1}Z_{gP1}^{\prime }\right){}^{\circ}\left(Z_{gP2}G_{P2}Z_{gP2}^{\prime }\right)\right)\right){}^{\circ}\left(Z_{E}Z_{E}^{\prime }\right)\sigma_{gEP1\times P2}^{2}\right)$

where $\sigma_{gEP1}^{2}$, $\sigma_{gEP2}^{2}$ and $\sigma_{gEP1\times P2}^{2}$ are the corresponding variance components for interaction terms between markers of inbreds and environments for the GCA (parent 1 and parent 2) and SCA (*P1* × *P2*) terms; *Z*_*E*_ is the corresponding incidence matrix for environments of order (276 × 4 = 1 104) × 4 for the case when the phenotypic information of inbreds was omitted and (309 × 4 = 1236) × 4 for the case when the calibration sets were augmented with phenotypic inbred data. The genetic effects of the genotypes derived from this model are particular to each environment.

The model components of M1-M3 are listed in **Table 1**, which shows how the models compare in terms of main and interaction effects. The main effect components are G_P1_, and G_P2_ [main effects of inbred markers accounting for paternal/maternal effects (GCA)], and G_P1__×__P2_ [interaction between inbred markers for paternal/maternal effects (SCA)], and the interaction effects are G_P1_ × E, and G_P2_ × E (interaction between inbred markers and environments), and G_P1__×__P2_ × E (interaction between SCA effects and environments). The described models (M1-M3) were fitted using the Bayesian Generalized Linear Regression (BGLR) R package (Perez-Rodriguez and de los Campos, 2014).

**Table 1:** Main and interaction components of three models (M1-M3) used for predicting crop yield performance.

Models/Components	Main effects				Interactions		
	E	G_P1_	G_P2_	G_P1×P2_	G_P1_ × E	G_P2_ × E	G_P1xP2_ × E
M1	**X**	**X**	**X**				
M2	**X**	**X**	**X**	**X**			
M3	**X**	**X**	**X**	**X**	**X**	**X**	**X**

The main effects are G_P1_, and G_P2_ [main effects of inbred markers accounting for paternal/maternal effects (GCA)], and G_P1__×__P2_ [interaction between inbred markers for paternal/maternal effects (SCA)]; the interaction effects are G_P1_ × E, and G_P2_ × E (interaction between inbred markers and environments), and G_P1__×__P2_ × E (interaction between SCA effects and environments).

### Prediction Schemes

For assessing model performance three CV schemes were implemented (CV2, CV1, and CV0) mimicking real life situations encountered in breeding programs. CV2 resembles incomplete field trials where some hybrids are tested in some environments but not in others, so the goal is to predict the performance of already tested hybrids in other environments in already observed environments where these hybrids have not been tested yet but others. The marker data and phenotypic information of the observed genotypes (hybrids, and hybrids and inbreds depending on the case) in training set are used for model calibration, the testing set is hypothetically composed only of maker data.

The prediction accuracy is calculated as the Pearson correlation coefficient between predicted and observed values within same environment. CV1 is the CV scheme where the goal is to predict the performance of lines that have not been tested in any of the environments. CV0 is the case where predictions are performed for a new environment, for which no phenotypic information has yet been collected. Here, the lines to be predicted were already tested in some other environments.

For CV2, phenotypes were randomly assigned to folds (5), each containing approximately 20% of the records. Prediction of each fold (one at a time) was conducted using the remaining folds (4) as the training set. After predicting each one of the folds, the predictions were integrated into a single vector and the Pearson correlation between predicted and observed values for each environment was computed. This procedure was repeated 50 times and the mean correlation was computed across replicates for each environment.

For CV1, a five-fold CV was also implemented, but with a change with respect to the previous scheme such that all phenotypic records of the same genotype were assigned to the same fold. Thus, no phenotypic records of the same genotype were encountered in two different folds. As in CV2, this procedure was repeated 50 times and the results were averaged.

CV0 does not require any random partitioning so it was implemented only once by deleting the phenotypic information of each environment (one at a time) and using the remaining environments as the training set for calibrating models. Then the correlation between the predicted and observed values for each environment was computed.

### Use of Phenotypic Information of Inbreds in Training Sets

For (*a*) the three CV schemes (CV2, CV1, and CV0), (*b*) the three models, and (*c*) the two genotyping platforms (C and T), two different scenarios for including phenotypic information of inbreds in calibration sets were considered. The first scenario ignores the phenotypic information of the inbreds when calibrating models, while the second one augments the training sets with the phenotypic information of the inbreds. For CV0, the phenotypic information of the inbreds tested in the target environment was also ignored for model calibration such that under this scheme no phenotypic information at all (hybrid and inbreed) from the target environment was used in the analysis. For including the inbred data in calibration sets, these were modeled as hybrids but with the same parent acting as parent 1 and parent 2. For this, the *X*_*P*1_, *X*_*P*2_ matrices were augmented with the molecular information of the same inbred in both matrices. Thus, hybrids were modeled when different parents were used in both matrices and inbreds when the same parent was used in both matrices.

## Results

### Descriptive Statistics

**Figure 1:**
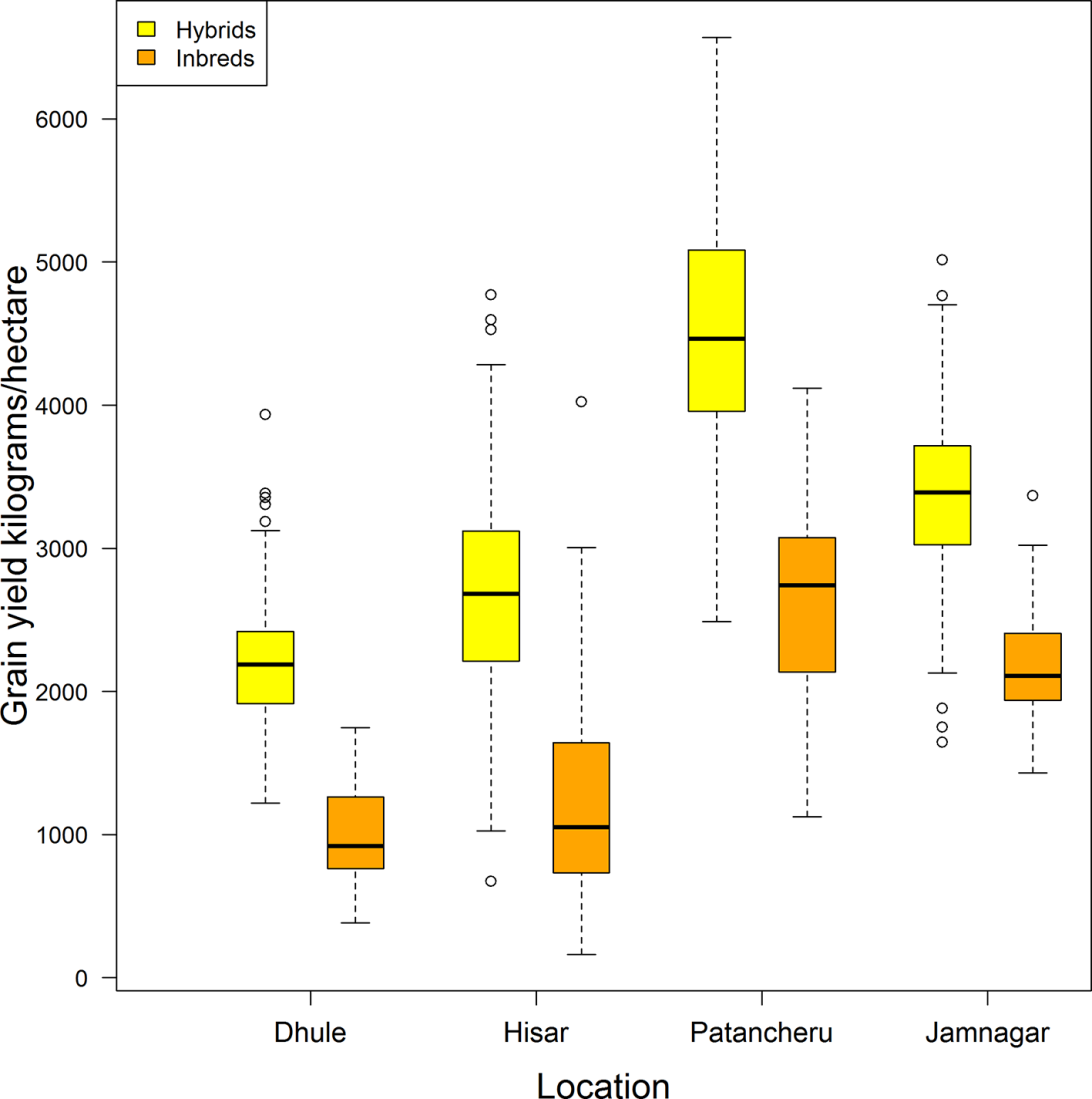
Distribution of grain yield for a pearl millet population comprising 276 hybrids (yellow) and 33 inbreds (orange) tested in four locations in India (all genotypes in all locations are included).

**Figure 1** displays a box-plot of the grain yield (in kilograms/hectare) of the 276 hybrids (in yellow) and the 33 inbreds (in orange) tested in four locations (Dhule, Hisar, Patancheru, and Jamnagar). All genotypes (hybrids and inbreds) were observed in all locations. Clear differences are apparent in yield performance among locations, and between hybrids and inbreds within locations and across locations. Patancheru had the highest average yield performance for hybrids (4,501.5 kg/ha) and inbreds (2,617.1 kg/ha). This location also showed the largest standard deviation [SD hybrids: 827.5 kg/ha ~18% of the mean location (ML); SD inbreds: 717.5 kg/ha ~27% of the ML]. Dhule returned the lowest average yield performance for both hybrids (2,197.1 kg/ha) and inbreds (1,004.2 kg/ha) with corresponding SDs of 394.7 kg/ha (~18% ML) and 339 kg/ha (~34% ML), respectively. These SDs were the smallest across locations.

### Phenotypic Correlations

**Table 2** shows phenotypic correlations among the four locations for four different scenarios. (a) Top-left sub-table, the upper off-diagonal contains the phenotypic correlation between hybrids tested in different locations. (b) Bottom-right sub-table, the upper off-diagonal contains the phenotypic correlation between inbreds tested in different locations. (c) The top-right panel contains the correlation between the hybrids with common parent 1 (P1, B-lines) and the corresponding inbred parents within and between locations. (d) The bottom-left panel contains the correlation between the hybrids with common parent 2 (P2, R-lines) and the corresponding inbred parents within and between locations. Because pearl millet hybrids are generated using cytoplasmic male sterility (CMS) systems, the female parent must be in a male sterile cytoplasm and the male parent must carry a restorer of fertility gene. Thus individual inbreds were only utilized as either a male parent or a female parent, such that inbreds can be divided into two disjoint groups (B-lines and R-lines).

**Table 2:** Sample phenotypic correlations within and between hybrids (H) and inbreds (I) tested in different locations.

		H				I			
		Dhule	Hisar	Patancheru	Jamnagar	Dhule	Hisar	Patancheru	Jamnagar
H	Dhule	1	0.243	0.391	0.160	0.018	0.053	0.046	0.036
	Hisar		1	0.322	0.236		0.363	0.173	0.157
	Patancheru			1	0.291	**P1 - B-lines**		-0.022	0.026
	Jamnagar				1				0.091
I	Dhule	0.069	0.203	0.263	0.185	1	0.290	0.472	0.418
	Hisar		0.351	0.114	0.068		1	0.578	0.358
	Patancheru	**P2 - R-lines**		0.405	0.326			1	0.555
	Jamnagar				0.199				1

The correlation between hybrids and inbreds was computed considering either parent 1 (P1—B-lines, 13 inbreds) or parent 2 (P2—R-lines, 20 inbreds) of hybrids as the common hub for connecting inbreds. P1 and P2 are disjoint groups of inbreds.

For the (a) scenario, locations Patancheru and Dhule showed the highest phenotypic correlation (0.391) between hybrids, while locations Jamnagar and Dhule exhibited the lowest value (0.160). Regarding the inbreds (i.e., b), locations Hisar and Patancheru (0.578) and Dhule and Hisar (0.290) returned the highest and the lowest phenotypic correlations. The highest and lowest within environments correlations between hybrids and inbreds when P1 was used as common hub for connecting both populations were shown in locations Hisar (0.363) and Patancheru (−0.022). Regarding the intra-location correlations, locations Hisar and Patancheru (0.173) and locations Jamnagar and Patancheru (0.026) returned the highest and lowest correlations. On the other hand, when P2 was used as the common hub, locations Patancheru (0.405) and Dhule (0.069) returned the highest and lowest correlations. Locations Patancheru and Jamnagar (0.326) and locations Hisar and Jamnagar (0.068) exhibited the highest and lowest intra-location correlations.

### Variance Components

**Table 3** contains the percentage of the total variability (and corresponding standard errors) explained by the model components for the two sequencing platforms (C and T). As expected, in all cases, the E term captured the largest amount of variability (48.3%–51.7%). The percentage of variability not explained by the main and interaction terms ranged between 21.9% and 39.8%, with the most comprehensive model reducing the residual variability the most.

**Table 3:** Percentage of variability (and corresponding standard errors) explained by main and interaction model terms for models M1, M2, and M3 using two sequencing platforms (C and T).

Platform	Models/Components	Main effects				Interaction effects			
		E	G_P1_	G_P2_	G_P1xP2_	G_P1_ x E	G_P2_ x E	G_P1xP2_ x E	R
**C**	M1	51.7 (31.6)	2.1 (1.0)	6.5 (2.5)					39.8 (1.8)
	M2	51.5 (34.9)	1.7 (0.9)	5.9 (2.3)	6.1 (1.6)				34.9 (1.8)
	M3	48.3 (30.8)	0.7 (0.5)	3.8 (2.1)	7.4 (1.9)	7.1 (2.2)	4.6 (1.6)	6.3 (1.5)	21.9 (1.7)
**T**	M1	50.8 (31.0)	1.8 (0.9)	8.2 (3.2)					39.2 (1.8)
	M2	51.0 (34.6)	1.5 (0.9)	7.3 (3.1)	5.0 (1.5)				35.3 (1.8)
	M3	49.1 (31.7)	0.7 (0.5)	4.7 (2.8)	7.0 (1.9)	4.9 (1.6)	5.1 (1.8)	5.2 (1.3)	23.4 (1.7)

The main effects are G_P1_ and G_P2_ [main effects of inbred markers accounting for paternal/maternal effects (GCA)], and G_P1__×__P2_ [interaction between inbred markers of parents 1 and 2 (SCA)]; the interaction effects are G_P1_ × E, and G_P2_ × E (interaction between inbred markers of parents 1 and 2 with environments), and G_P1__×__P2_ × E (interaction between SCA effects and environments); R stands for residual term.

Under the C platform, the terms G_P1_ and G_P2_ in M1 explained 8.6% (2.1% + 6.5%) of the total variability, where G_P2_ (6.5%) explained three times more variability than G_P1_ (2.1%). In M2, the SCA term (G_P1__×__P2_, 6.1%) captured similar levels of variability than the combined GCA components (1.7% and 5.9%) providing strong evidence of the importance of modeling this term.

When the interactions of the GCA and SCA terms with environments were included in M3, combined these terms explained 18% (7.1, 4.6, and 6.3%) of the variability. Similar patterns were observed for the T platform.

### Predictive Ability

**Figure 2:**
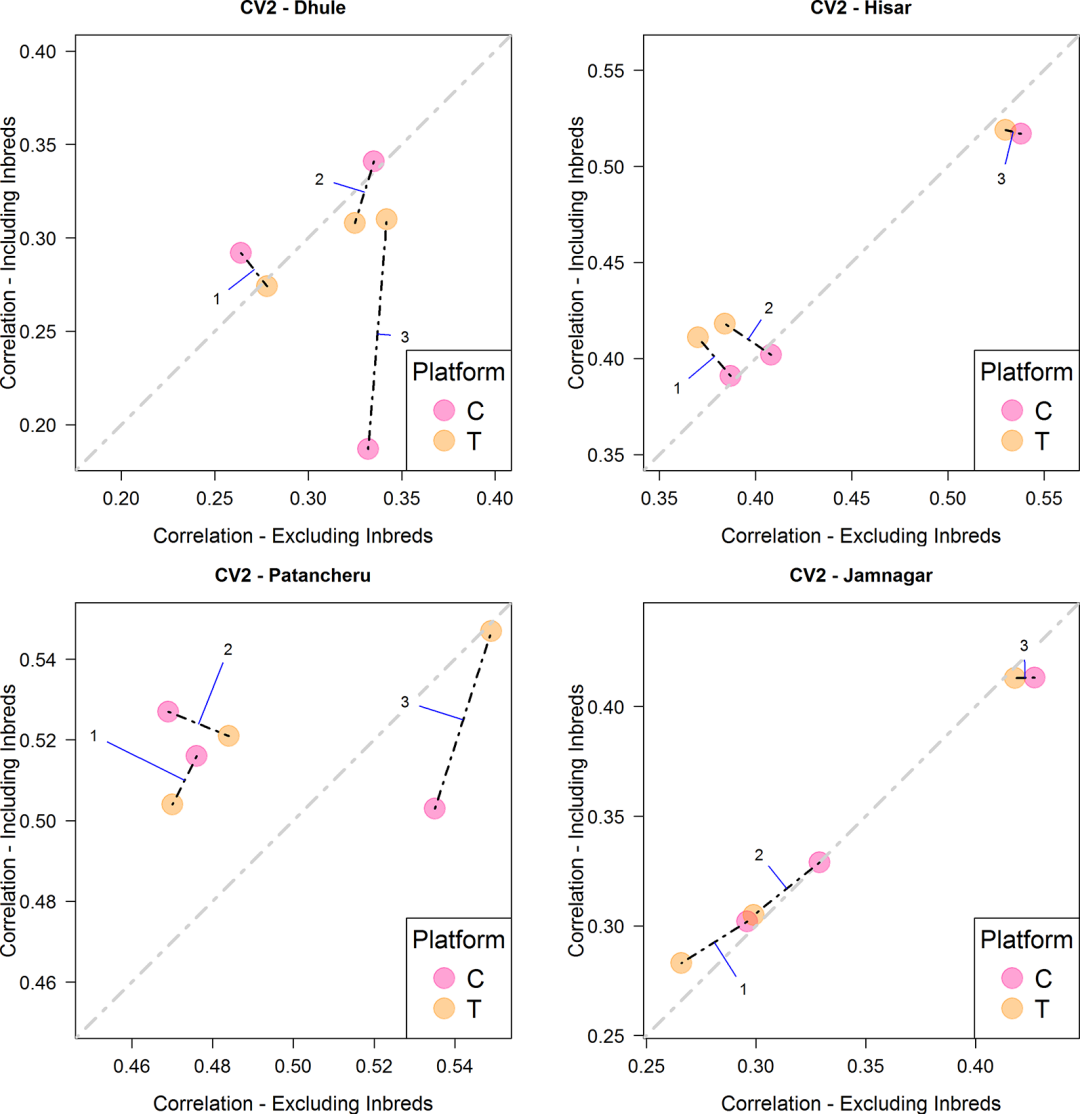
Comparison of the average predictive ability (correlation between true and predicted grain yield values) of models M1-M3 for the cross-validation scenario CV2 (prediction of tested hybrids in observed environments) using genotyping platforms C or T when inbred information is included versus when no inbred information is included. The four panels represent the four different environments (E1: Dhule, E2: Hisar, E3: Patancheru, E4: Jamnagar). The models are M1: G_P1_ + G_P2_; M2: G_P1_ + G_P2_ + G_P1__×__P2_; M3: G_P1_ + G_P2_ + G_P1__×__P2_ + G_P1_ × E + G_P2_ × E + G_P1__×__P2_ × E, where G_P1_, and G_P2_ are the genetic main effects of the parental inbreds P1 and P2, respectively (GCA components), G_P1__×__P2_ is the interaction effect of the parental inbreds P1 and P2 (SCA component), E is the environment, and “×” represents the interaction between the specified terms.

**Figure 2** and **Table 4** summarize the results derived from the CV2 scheme, which considers the problem of predicting incomplete field trials. The four panels correspond to the four environments/locations used in this study. The *x-axis* correspond to the obtained correlation between predicted and observed values when no phenotypic information of inbreds was used for model calibration. On the other hand, the *y-axis* shows the correlation for the case when phenotypic information of the inbreds was added to calibration set. The diagonal line indicates the case where there are no differences in predictive ability by including information of the inbreds in the training set. Values above the line indicate that the models including phenotypic information of the inbreds perform better. Same models tested under different platforms (C is represented by blue colored dots and T by orange colored dots) are connected with a black dashed line. In general, the T platform produced better results than the C platform, especially with the most comprehensive model (i.e., M3). Consistently, the most complex model also returned the highest correlation in most of the environments except in location Dhule where the C platform had a significantly reduced predictive ability for M3. Regarding the use of inbred data in the calibration sets, a slight improvement was shown for M1-M2 but not always for M3. In location Hisar, the inbred data improved the results for M1-M2; however, M3 produced the best results by far. A similar pattern was observed for Patancheru. Jamnagar did not show significant differences between the two different ways of calibrating models but again M3 outperformed the results despite the use of inbred data.

**Table 4:** Mean predictive ability and corresponding SDs for models M1, M2, and M3 using two sequencing platforms (C and T) for different cross-validation (CV) schemes and two ways for composing calibration sets (including or not phenotypic inbred data).

			CV2						CV1						CV0		
		Environment	M1		M2		M3		M1		M2		M3		M1	M2	M3
			Mean	SD	Mean	SD	Mean	SD	Mean	SD	Mean	SD	Mean	SD	Mean	Mean	Mean
**Inbreds not Included**	C	Dhule	0.264	0.023	0.335	0.022	0.332	0.020	0.218	0.024	0.197	0.033	0.116	0.044	0.288	0.340	0.412
		Hisar	0.387	0.023	0.408	0.021	0.538	0.011	0.367	0.017	0.326	0.027	0.460	0.028	0.334	0.380	0.165
		Patancheru	0.476	0.029	0.469	0.024	0.535	0.021	0.453	0.026	0.406	0.035	0.449	0.023	0.426	0.479	0.490
		Jamnagar	0.296	0.019	0.329	0.017	0.427	0.021	0.278	0.017	0.266	0.013	0.360	0.027	0.269	0.294	0.328
	T	Dhule	0.278	0.029	0.325	0.026	0.342	0.016	0.236	0.021	0.221	0.019	0.159	0.023	0.306	0.336	0.371
		Hisar	0.370	0.024	0.384	0.023	0.530	0.016	0.349	0.019	0.318	0.022	0.478	0.026	0.277	0.314	0.359
		Patancheru	0.470	0.026	0.484	0.027	0.549	0.015	0.446	0.023	0.418	0.026	0.485	0.030	0.417	0.469	0.508
		Jamnagar	0.266	0.021	0.299	0.021	0.418	0.018	0.245	0.018	0.244	0.019	0.364	0.019	0.231	0.265	0.322
**Inbreds Included**	C	Dhule	0.292	0.015	0.341	0.014	0.187	0.029	0.260	0.017	0.260	0.018	0.102	0.014	0.298	0.337	0.222
		Hisar	0.391	0.021	0.402	0.018	0.517	0.012	0.377	0.018	0.359	0.015	0.481	0.020	0.302	0.328	0.329
		Patancheru	0.516	0.022	0.527	0.020	0.503	0.026	0.497	0.019	0.482	0.019	0.478	0.021	0.451	0.493	0.234
		Jamnagar	0.302	0.018	0.329	0.017	0.413	0.021	0.284	0.017	0.297	0.016	0.383	0.020	0.271	0.300	0.325
	T	Dhule	0.274	0.014	0.308	0.014	0.310	0.015	0.240	0.016	0.246	0.016	0.209	0.017	0.283	0.310	0.340
		Hisar	0.411	0.020	0.418	0.018	0.519	0.017	0.400	0.018	0.388	0.018	0.489	0.021	0.328	0.345	0.521
		Patancheru	0.504	0.021	0.521	0.020	0.547	0.015	0.486	0.017	0.481	0.018	0.521	0.019	0.444	0.473	0.411
		Jamnagar	0.283	0.017	0.305	0.015	0.413	0.015	0.263	0.015	0.268	0.014	0.379	0.021	0.237	0.263	0.331

For CV2 and CV1, 50 random fivefold random partitions were considered while for CV0 the leave one environment out scheme was implemented. The underlined numbers indicate the model that returned the highest correlation for each environment within each cross-validation scheme.

**Figure 3:**
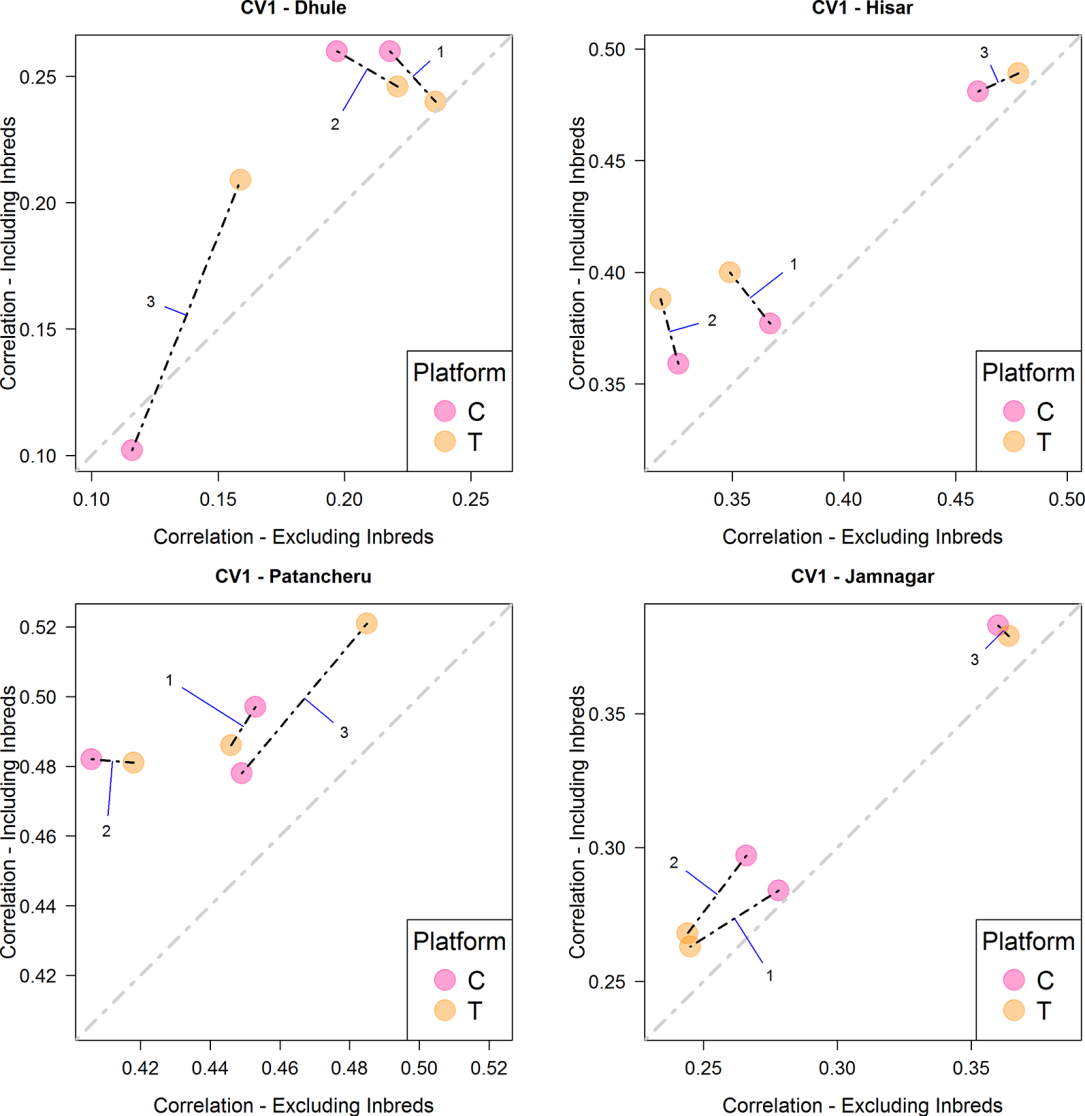
Comparison of the average predictive ability (correlation between true and predicted grain yield values) of models M1-M3 for the cross-validation scenario CV1 (prediction of untested hybrids in observed environments) using genotyping platforms C or T when inbred information is included versus when no inbred information is included. The four panels represent the four different environments (E1: Dhule, E2: Hisar, E3: Patancheru, E4: Jamnagar). The models are M1: G_P1_ + G_P2_; M2: G_P1_ + G_P2_ + G_P1__×__P2_; M3: G_P1_ + G_P2_ + G_P1__×__P2_ + G_P1_ × E + G_P2_ × E + G_P1__×__P2_ × E, where G_P1_, and G_P2_ are the genetic main effects of the parental inbreds P1 and P2, respectively (GCA components), G_P1__×__P2_ is the interaction effect of the parental inbreds P1 and P2 (SCA component), E is the environment, and “×” represents the interaction between the specified terms.

When predicting new genotypes in tested environments (CV1), platform T was consistent and produced the best results with M3 (**Figure 3** and **Table 4**). These models outperformed the others in Hisar, Patancheru, and Jamnagar. Dhule was the only location where M1-M2 returned better results than M3. In most cases, the inclusion of inbred data improved predictive ability compared with the case when this information was not considered in the calibration sets.

**Figure 4:**
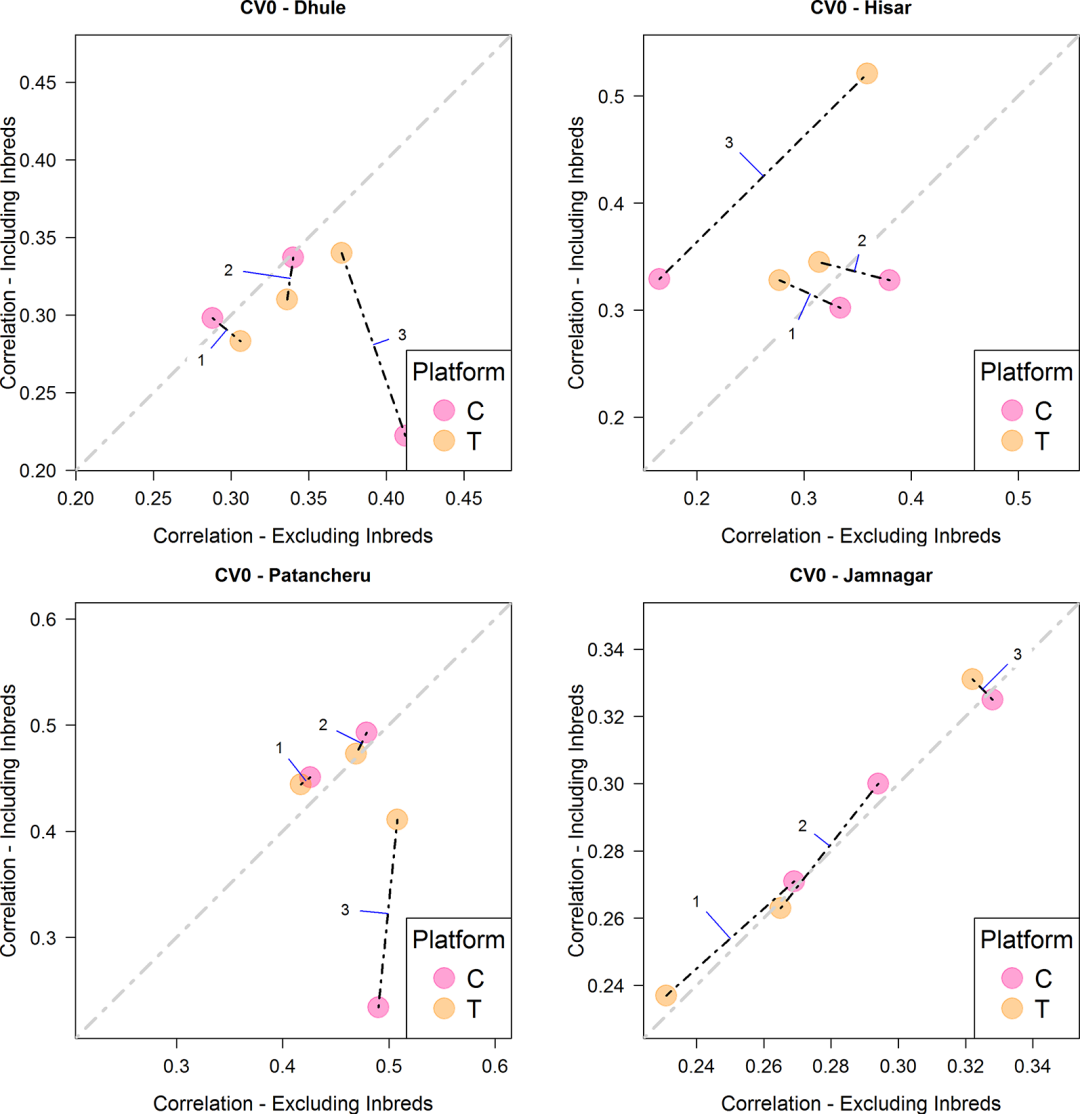
Comparison of the average predictive ability (correlation between true and predicted grain yield values) of models M1-M3 for the cross-validation scenario CV0 (prediction of tested hybrids in unobserved environments) using genotyping platforms C or T when inbred information is included versus when no inbred information is included. The four panels represent the four different environments (E1: Dhule, E2: Hisar, E3: Patancheru, E4: Jamnagar). The models are M1: G_P1_ + G_P2_; M2: G_P1_ + G_P2_ + G_P1__×__P2_; M3: G_P1_ + G_P2_ + G_P1__×__P2_ + G_P1_ × E + G_P2_ × E + G_P1__×__P2_ × E, where G_P1_, and G_P2_ are the genetic main effects of the parental inbreds P1 and P2, respectively (GCA components), G_P1__×__P2_ is the interaction effect of the parental inbreds P1 and P2 (SCA component), E is the environment, and “×” represents the interaction between the specified terms.

When the objective was the prediction of tested hybrids in unobserved environments (CV0), the T platform returned the best results, especially with M3, similarly to the CV2 scheme (**Figure 4** and **Table 4**). In this case, for locations Hisar and Jamnagar, the inclusion of inbred data showed significant advantages in predictive ability. In Dhule and Patancheru, no clear advantages were apparent with the models M1-M2, and with model M3 the predictive ability was slightly reduced. However, similarly than in the previous schemes M3 performed better or slightly better than M1 and M2.

## Discussion

In this study, we found that the integration of phenotypic information from parental inbreds improves the predictive ability of the tested models in several cases. Especially in environments where the phenotypic correlation between inbreds and hybrids was moderate (bottom left and top right panels of **Table 2**). In the Hisar field trials, the yield of the tested hybrids was moderately correlated with each parent; r = 0.363 for Parent 1 (seed parent—B-lines) and and r = 0.351 for Parent 2 (pollinator parent—R-lines). In the Patancheru field trials, yield for the tested hybrids was moderately correlated with Parent 2 (r = 0.405), but exhibited zero correlation with Parent 1 (r = −0.022).

The inclusion of the G×E interaction term improved the predictive ability of the main effect models in three out the four locations (except in Dhule). In this case the inclusion of the G×E component was beneficial for improving predictive ability because some of the environments exhibited low to moderate phenotypic correlation among hybrids, inbreds, and between hybrids and inbreds (connected *via* one or both of the parental inbreds). For example, the phenotypic correlations between hybrid yields measured in in Hisar and hybrid yields recorded at the other three locations were 0.243, 0.391, and 0.160. Similarly, the correlations between inbred yields at this location and inbred yields at the other three locations were moderate (0.290, 0.472, and 0.418). The correlations between hybrid yields in Hisar and inbred yields at other locations were also low to moderate for P1 (r = 0.053 [Dhule], 0.173 [Patancheru], and 0.157 [Jamnagar]) and P2 (r = 0.203 [Dhule], 0.114 [Patancheru], and 0.068 [Jamnagar]).

Under the CV2 scheme, including phenotypic inbred data in the calibration sets improved predictive ability for hybrid performance in Hisar and Patancheru field trials. These two locations showed the highest phenotypic correlations between hybrids and parental inbreds (0.363 and 0.351 for Hisar and −0.022 and 0.405 for Patancheru). Dhule (0.018 and 0.069) and Jamnagar (0.091 and 0.199) showed smaller correlations for both parental inbreds.

As expected, predictive ability for CV1 was lower than for CV2, since no phenotypic information of the hybrids being evaluated was used to calibrate the models. Hence, the predictive ability of the models rely solely on genomic relationships/connectedness between the calibration and testing sets in CV1 scheme. In this case, the inclusion of the phenotypic information from inbreds improved predictive ability, as is evident from the fact that most dots appear above the diagonal line in **Figure 3**.

When predicting tested genotypes in unobserved environments (CV0), predictive ability mostly relies on the phenotypic correlation between those environments in the calibration set with the target environment. In this case, Hisar and Jamnagar showed the best results when phenotypic inbred data from the observed environments was added to the calibration sets. The phenotypic correlations between the inbreds tested in these environments with respect to other environments were 0.290, 0.578, and 0.358 for Hisar and 0.418, 0.358, and 0.555 for Jamnagar (**Table 2**).

The interaction model (M3) outperformed the main effects models in most cases. Usually these appear on the right side (*x-axis*, when inbred data do not aid predictive ability) or at the top (*y-axis*, when inbred data aid predictive ability) or both (top right) in **Figures 2**–**4**. In the CV2 scheme, the interaction model clearly improved upon the main effect models for predicting hybrid performance in Hisar (24–43%), Patancheru (−5–17%), and Jamnagar (25–46%). In Dhule under the T platform the interaction model improved the others between 1 and 23%. Negative improvements were observed under the C platform. Similarly, under both platforms (C and T) in the CV1, the interaction model outperformed the main effects models in the same locations (up to 50% in Jamnagar and Hisar), except in Dhule were the predictive ability was reduced by half. For CV0 scheme, M3 also outperformed M1 and M2 between 10 and 45% except when the C platform was combined with phenotypic information from inbreds where the predictive ability was reduced between 25 and 34%. In the case of CV0 we are predicting the performance of lines in a new environment, thus we do not have any information about the performance of these lines or any other lines in the target environment. However, we borrow information of the performance of the same lines in other environments, and also of other lines in other environments. In absence of weather data the success of this method and particularly of M3 would depends partly on whether the environmental conditions in the unobserved environment fall within the range of conditions of the environments used as training sets.

When comparing the usefulness of the different sequencing data for prediction purposes, the T platform produced higher prediction accuracy than C platform in most cases for same model-training set configurations. For CV2, the T platform returned the best results, particularly for model M3. Especially, when predicting hybrid performance in yield trials at Patancheru. For the other locations, no clear differences between these platforms were observed with this model and mixed result were obtained for M1 an M2. The use of inbred phenotypic information improved predictive ability in Patancheru and Hisar for M1 and M2; however, these result were never better than those derived from M3 which did not show improvements by augmenting the calibration sets.

Using the CV1 cross-validation scheme, in Patancheru and Hisar, the T platform returned the best results when combined with phenotypic inbred data under M3. No clear differences/patterns were observed in Dhule and Jamnanar. The T platform showed significant improvement with respect to platform C for making hybrid performance predictions in Dhule with model M3, while for the other models, the C platform performed slightly better than T platform and than M3 as well. In Jamnagar, platforms T and C performed equivalently for M3, while for the other models (M1-M2) platform C provided greater prediction accuracy than platform T but these result were never better than those derived from M3. Under this CV scheme, despite the model and platform used in the analysis the predictive ability was improved in 23 out of the 24 cases when phenotypic information of the inbreds was added in calibration sets.

Using the CV0 cross validation scheme, results from the T platform outperformed significantly those from the C platform in Hisar under M3. In Patancheru, the T and C platforms performed similarly for M1-M3. In Dhule, the C platform performed slightly better than the T platform for model M3, and no differences were observed for M1-M2. Under this scheme, the predictive ability was significant improved when phenotypic data from the inbreds was included in calibration sets for M3 in Hisar while for Jamnagar these improvements were smaller. For Patancheru, M1 and M2 slightly improved the results and in Dhule no advantages were observed by adding the phenotypic inbred data.

## Conclusions

GS is a promising tool that enables us to study the crop performance of genotypes yet to be observed. The use of the GCA and SCA terms makes it possible to model and predict the performance of hypothetical genotypes. By combining these two concepts, we can increase our power to search for and identify superior cultivars (hybrids).

In this study, we observed improvements in predictive ability by including phenotypic information of parental inbreds in the calibration datasets, especially when predicting untested or target hybrids to be created. While these improvements were often modest but not always, it should be noted that one explanation is that the number of inbred genotypes (33) used for model calibration included only about 1/10 of all the hybrid genotypes (276).

In general, including the interaction terms in the models in addition to the main effect terms, significantly improved prediction accuracy. Finally, we compared the accuracy provided by two different genotyping platforms, and found that the use of the T platform provided better or the same accuracy of hybrid performance prediction as the C platform using the most successful model (M3).

## Data Availability Statement

Publicly available datasets were analyzed in this study. This data can be found here: https://doi.org/10.6084/m9.figshare.5969230, https://doi.org/10.6084/m9.figshare.5566843.

## Author Contributions

DJ conceived the work, performed the data analysis, discussed the different aspects of the genotype-by-environment interaction in the Genomic Selection context, and wrote the first draft of the paper. RH conceived the work, supervised the data analysis, discussed the key aspects of Genomic Selection, and wrote the paper. ZL provided the phenotypic and molecular marker data used in the analysis, retrieved the information about the different sequencing technologies, and wrote the paper. SG provided the phenotypic and molecular marker data used in the analysis, contribute in the discussion and conclusions, and wrote the paper. JS provided the phenotypic and molecular marker data used in the analysis, and wrote the paper. JC discussed the different aspects of the genotype-by-environment interaction in the Genomic Selection context, discussed the different manners for modeling hybrids, and wrote the paper.

## Funding

This work is supported by award 2016-67013-24613 from the USDA National Institute of Food and Agriculture to JS.

## Conflict of Interest

The authors declare that the research was conducted in absence of any commercial or financial relationship that could be construed as a potential conflict of interest.

The reviewer TH and handling Editor declared their shared affiliation.

## References

[B1] Acosta-Pech R. Crossa J. de los Campos G. Teyssèdre S. Claustres B. (2017). Genomic models with genotype x environment interaction for predicting hybrid performance: an application in maize hybrids. Theor. Appl. Genet. 130, 1431. 10.1007/s00122-017-2898-0 28401254

[B2] Baird N. A. Etter P. D. Atwood T. S. Currey M. C. Shiver A. L. Lewis Z. A. (2008). Rapid SNP discovery and genetic mapping using sequenced RAD markers. PloS One 3 (10), p. e3376. 10.1371/journal.pone.0003376 18852878 PMC2557064

[B3] Basnet B. R. Crossa J. Pérez-Rodríguez P. Manes Y. Singh R. Rosyara U. (2018). Hybrid wheat prediction using genomic, pedigree and environmental covariables interaction models. Plant Genome 12, 180051. 10.3835/plantgenome2018.07.0051 PMC12810112 30951082

[B4] Bernardo R. (1994). Prediction of maize single-cross performance using RFLPs and information from related hybrids. Crop Sci. 34, 20–25. 10.2135/cropsci1994.0011183X003400010003x

[B5] Browning B. L. Zhou Y. Browning S. R. (2018). A one-penny imputed genome from next generation reference panels. Am. J. Hum. Genet. 103 (3), 338–348. 10.1016/j.ajhg.2018.07.015 30100085 PMC6128308

[B6] Kadam D. C. Potts S. M. Bohn M. O. Lipka A. Lorenz A. J. (2016). Genomic prediction of single crosses in the early stages of maize hybrid breeding pipeline. Genes Genom. Genet. 6, 3443–3453. 10.1534/g3.116.031286 PMC5100843 27646704

[B7] Kim C. Guo H. Kong W. Chandnani R. Shuang L. S. Paterson A. H. (2016). Application of genotyping by sequencing technology to a variety of crop breeding programs. Plant Sci. 242, 14–22. 10.1016/j.plantsci.2015.04.016 26566821

[B8] Li J. Chen G. B. Rasheed A. Li D. Sonder K. Zavala Espinosa C. (2019). Identifying loci with breeding potential across temperate and tropical adaptation *via* EigenGWAS and EnvGWAS. Molecular ecology. 10.1111/mec.15169 PMC6851670 31287919

[B9] Liang Z. Gupta S. K. Yeh C.-T. Zhang Y. Ngu D. W. Kumar H. (2018). Phenotypic data from inbred parents can improve genomic prediction in pearl millet hybrids. G3: Genes, Genomes, Genetics 8, 2513–2522. 10.1534/g3.118.200242 29794163 PMC6027876

[B10] Massman J. M. Gordillo A. Lorenzana R. E. Bernardo R. (2013). Genomewide predictions from maize single-cross data. Theor. Appl. Genet. 126, 13–22. 10.1007/s00122-012-1955-y 22886355

[B11] Miller M. R. Dunham J. P. Amores A. Cresko W. A. Johnson E. A. (2007). Rapid and costeffective polymorphism identification and genotyping using restriction site associated dna (rad) markers. Genome Res. 17, 240–248. 10.1101/gr.5681207 17189378 PMC1781356

[B12] Ott A. Liu S. Schnable J. C. Yeh C. T. E. Wang K. S. Schnable P. S. (2017). tGBS^®^ genotyping-by-sequencing enables reliable genotyping of heterozygous loci. Nucleic Acids Res. 45 (21), e178–e178. 10.1093/nar/gkx853 29036322 PMC5716196

[B13] Perez-Rodriguez P. de los Campos G. (2014). Genome-wide regression & prediction with the BGLR statistical package. Genetics 198, 483–495. 10.1534/genetics.114.164442 25009151 PMC4196607

[B14] Technow F. Schrag T. A. Schipprack W. Bauer E. Simianer H. Melchinger A. E. (2014). Genome properties and prospects of genomic prediction of hybrid performance in a breeding program of maize. Genetics 197 (4), 1343–1355. 10.1534/genetics.114.165860 24850820 PMC4125404

[B15] Varshney R. K. Shi C. Thudi M. Mariac C. Wallace J. Qi P. (2017). Pearl millet genome sequence provides a resource to improve agronomic traits in arid environments. Nat. Biotechnol. 35 (10), 969. 10.1038/nbt.3943 28922347 PMC6871012

[B16] Zheng Z. Sun Z. Fang Y. Qi F. Liu H. Miao L. (2018). Genetic diversity, population structure, and botanical variety of 320 global peanut accessions revealed through tunable genotyping-by-sequencing. Sci. Rep. 8 (1), 14500. 10.1038/s41598-018-32800-9 30266974 PMC6162295

